# Ketamine induces endoplasmic reticulum stress in rats and SV-HUC-1 human uroepithelial cells by activating NLRP3/TXNIP aix

**DOI:** 10.1042/BSR20190595

**Published:** 2019-10-18

**Authors:** Lingjuan Cui, Xiaoyan Jiang, Chengjun Zhang, Danxia Li, Shengqiang Yu, Fengchun Wan, Yue Ma, Wei Guo, Zhengfei Shan

**Affiliations:** 1 Department of Blood Purification, The Affiliated Yantai Yuhuangding Hospital of Qingdao University, Yantai, China; 2 Department of Organ Transplantation, The Affiliated Yantai Yuhuangding Hospital of Qingdao University, Yantai, China; 3 Department of Urology, The Affiliated Yantai Yuhuangding Hospital of Qingdao University, Yantai, China; 4 Department of Urology, The Affiliated Hospital of Qingdao University, Qingdao, China

**Keywords:** apoptosis, Ketamine, NOD-like receptor 3/Thioredoxin-interacting protein, SV-HUC-1 cells

## Abstract

Many clinical studies have been conducted on ketamine-associated cystitis. However, the underlying mechanisms of ketamine-associated cystitis still remain unclear. Bladder tissues of rats were stained by Hematoxylin and Eosin (HE). The viability of human uroepithelial cells (SV-HUC-1 cells) was determined by cell counting kit-8 (CCK-8). Apoptosis and reactive oxygen species (ROS) were examined by flow cytometry. Additionally, the expressions of tumor necrosis factor-α (TNF-α), interleukin-6 (IL-6), IL-1β and IL-18 were respectively determined by reverse transcription quantitative (RTq)-PCR and enzyme-linked immunosorbent assay (ELISA). The mRNA and protein levels of B-cell lymphoma/leukemia-2 (Bcl2), Bcl-2-associated X protein (Bax), cleaved caspase 3, glucose-regulated protein 78 (GRP78), CCAAT/enhancer binding protein homologous protein (CHOP), NOD-like receptor 3 (NLRP3), thioredoxin-interacting protein (TXNIP), Catalase and MnSOD were examined by RT-qPCR and Western blot. Small interfering RNA target TXNIP transfection was performed using Lipofectamine™ 2000. We found that ketamine effectively damaged bladder tissues of rats and promoted apoptosis through regulating the expression levels of GRP78, CHOP, Bcl-2, Bax and cleaved Caspase-3 proteins *in vivo* and *in vitro*. NLRP3 inflammatory body and TXNIP were activated by ketamine, which was supported by the changes in TNF-α, IL-6, IL-1 and IL-18 *in vivo* and *in vitro*. Furthermore, knocking down TXNIP reversed the effects of ketamine on apoptosis and NLRP3 inflammatory body in SV-HUC-1 cells. Meanwhile, the changes of Catalase and MnSOD showed that ROS was enhanced by ketamine, however, such an effect was ameliorated by down-regulation of TXNIP in SV-HUC-1 cells. Ketamine promoted cell apoptosis and induced inflammation *in vivo* and *in vitro* by regulating NLRP3/TXNIP aix.

## Introduction

Ketamine, which is a derivative of N-1-phenycyclohexy-piperidine (PCP), is a non-competitive antagonist of glutamate N-methyl-d-aspartate (NMDA) receptor. As ketamine inhibits the thalamic–neocortical system, selectively blocks pain and has an analgesic pharmacological effect, it is considered as an effective anesthetic [1–3]. Studies have shown that long-term abuse of ketamine often causes damage to acute urological and medical issues, and one of the most common complications is ketamine-associated cystitis [4–6]. However, the underlying mechanism of ketamine-associated cystitis progression remains to be elucidated.

Our previous research findings demonstrated that ketamine enhanced the production of reactive oxygen species (ROS) in human ureteral epithelial cells (SV-HUC-1) cells [7]. ROS overactivation activates the development of ERS and leads to the accumulation of unfolded proteins, which can be isolated from the three sensors of ERS to activate endoplasmic reticulum (ER) chaperone glucose-regulated protein 78 (GRP78) [8]. Therefore, a rapid up-regulation of GRP78 is considered as the most sensitive marker of ER stress. CCAAT/enhancer binding protein homologous protein (CHOP) is an important signaling molecule for pro-apoptosis, and ER stress-specific transcription factor, which has a low expression under normal conditions but a high expression under ER stress [9]. Thus, CHOP is considered as a marker of ER stress. Caspase could control the occurrence and progression of apoptosis by causing nuclear shrinkage and DNA segmentation to form apoptosis through cleaving protein kinases, nucleases and cytoskeleton [10,11]. Among them, caspase3 is the most important effector protease in the caspase cascade [12]. Bcl-2-associated X protein (Bax)/B-cell lymphoma/leukemia-2 (Bcl2) has been recognized as a protein associated with apoptosis [13]. Ketamine causes liver and neuronal apoptosis via Bax/Bcl2-mitochondrial-caspase protease pathway [14,15]. It is speculated that the Bax/Bcl2-mitochondrial-caspase protease pathway may be one of the related mechanisms underlying ketamine-induced apoptosis of bladder epithelial cells.

Recent researches have demonstrated that inflammation could induce ROS, leading to oxidative stress and may result in bladder dysfunction [16,17]. As an endogenous inhibitor of thioredoxin, thioredoxin-interacting protein (TXNIP) is an important antioxidant reductive protein and anti-apoptotic protein in cells [18]. Studies have shown that TXNIP activated NOD-like receptor 3 (NLRP3) inflammatory pathway under oxidative stress, thus causing the secretion of the pro-inflammatory cytokines interleukin (IL)-1β and IL-18 [19,20]. In recent years, it was found that NLRP3 inflammation was involved in the progression of cystitis [21,22], therefore, targeting the TXNIP/NLRP3 signal axis may be an effective therapeutic target for treating ketamine-associated cystitis.

Our study provides a better understanding ofn the underlying mechanisms of ketamine in ER stress and TXNIP/NLRP3 *in vivo* and *in vitro* as well as target strategy for treating ketamine-associated cystitis.

## Materials and methods

### Rats and ketamine treatment

Adult male Wistar rats (180–200 g) were purchased from the SLAC Laboratory Animal Co., Ltd. (Shanghai, China). The rats were kept in a specific pathogen-free (SPF) environment at room temperature (25 ± 2°C) in 60–80% humidity under a 12-h/12-h light/dark cycle, and free access to food and water was provided. The rats were randomly divided into four groups, which were control group, saline group (NC), low-dose group (L-KET, 5 mg/kg) and high-dose group (H-KET, 50 mg/kg), with six rats in each group. The rats in the experimental group were intraperitoneally injected with ketamine or saline at the same volume at 3:00 p.m. everyday for 3 months. All animal experiments were carried out at The Affiliated Yantai Yuhuangding Hospital of Qingdao University and approved by Qingdao University Animal Ethics Committee (QD2573466).

### Hematoxylin and Eosin staining

A small section was cut from bladder tissue of the rat by using microtome. The specimen was fixed with a 10% paraformaldehyde solution for more than 48 h, conventionally dehydrated and paraffin-embedded to prepare a 5-micron tissue section. The slices were baked in a 68°C incubator for 1–2 h and then placed in xylene for 30 min for three times to be dewaxed. Next, the sections were placed in 100, 95, 85 and 75% gradient alcohol for 5 min to be hydrated. After washing the slices for 2 min by tap water, the sections were stained by Hematoxylin for 10 min and by Eosin for 30 s. The sections were then infiltrated with xylene for 5 s, sealed by neutral gum and observed under a microscope (Olympus, Japan).

### Cells and culture

SV-HUC-1 cells used in the present study were purchased from the American Type Culture Collection (ATCC, Wiltshire, U.S.A.) and were grown in RPMI 1640 medium containing 10% fetal bovine serum (FBS, Gibco, Carlsbad, CA, U.S.A.) in a humidified atmosphere with 5% CO_2_ at 37°C. The SV-HUC-1 cells were treated with 0.01, 0.1 and 1 mmol/l concentrations of ketamine and then the SV-HUC-1 cells were co-treated by 1 mmol/l ketamine with or without small interfering RNA of TXNIP or NC RNA.

### Cell counting kit-8

Ketamines (0.01, 0.1 and 1 mmol/l) were used to treat the SV-HUC-1 cells for 24, 48 and 72 h, and cell viability was detected by cell counting kit-8 (CCK-8). To be more specific, the cells were digested with trypsin, adjusted to 1000 cells/well and placed in a 96-well culture plate at a volume of 100 μl/well. The medium in each well to be tested was washed away, and 10 μl of CCK-8 reagent was added to the well and incubated at 37°C with 5% CO_2_ for 2 h. A microplate reader (Bio-Rad Laboratories, Inc., Hercules, CA, U.S.A.) was used to determine the OD at an absorbance of 450 nm in each well in different cell groups.

### Enzyme-linked immunosorbent assay

Cells were treated with ketamine (0.01, 0.1 and 1 mmol/l) or 1 mmol/l ketamine in combination with siRNA TXNIP or NC siRNA. Protein was isolated by RIPA (Cell Signaling Technology, Inc., Danvers, MA, U.S.A.), and BCA Protein Assay Kit (Pierce) was used to measure the concentrations of the proteins. The levels of IL-1β and IL-18 were determined using corresponding enzyme-linked immunosorbent assay (ELISA) (MD SpectraMax M5; Molecular Devices, U.S.A.) following the manufacturer’s instructions.

### Apoptosis

Apoptosis detection kit (BD Biosciences Medical Devices Shanghai Co., Ltd., Shanghai, China) was used to detect SV-HUC-1 cells apoptosis. The SV-HUC-1 cells (5 × 10^5^ cells/well) were seeded in six-well plates to reach 85% cell density. Ketamine at concentrations of 0.01, 0.1 and 1 mmol/l were used to treat SV-HUC-1 cells, and 1 mmol/l ketamine-treated SV-HUC-1 cells were treated by siTXNIP, cultured in serum-free medium for 5 h and the medium was replaced. After 48 h of culture, the cells were digested, centrifuged and washed twice with phosphate-buffered saline (PBS). A total of 100 μl of 1× Annexin-V Binding Buffer and 5 μl FITC-labeled Annexin-V (20 μg/ml) were added to the cells, which were kept at room temperature for 20 min. Next, the cells were added with 5 μl of PI (50 μg/ml), held for 5 min and 400 μl of binding buffer was then added. A flow cytometer (BD Bioscience, Shanghai, China) was used to analyze cell apoptosis.

### Reverse transcription quantitative polymerase chain reaction

After the transfection, SV-HUC-1 cells were incubated in an incubator for 48 h, and total RNA from SV-HUC-1 cells was extracted using TRIzol® reagent (Thermo Fisher Scientific, Inc.). A NanoDrop™ 2000 spectrophotometer (Thermo Fisher Scientific, Inc.) was conducted to quantify RNA (an A260/A280 ratio between 1.8 and 2.0 was required for generating cDNA). Oligo-dT or stem-loop reverse transcriptase primers (Takara Bio, Inc., Otsu, Japan) were used to obtain cDNA, and the reaction conditions were set as follows: at 42°C for 60 min, 70°C for 5 min and preserved at 4°C. qPCR was performed by the SYBR® Premix ExTaq™ II (Takara Bio Inc.) using real-time PCR Detection System (ABI 7500, Life Technology, U.S.A.). PCR conditions were set as follows: a pretreatment at 95°C for 10 min, followed by 40 cycles at 94°C for 15 s, at 60°C for 1 min, finally at 60°C for 1 min and preserved at 4°C. The 2^−ΔΔ*C*^_q_ method was used to process the data. The primers used in this experiment are listed in Table 1.

**Table 1:** Primers for RT-qPCR (R = Rat, H = Human)

Genes	Forward	Reverse
*GRP78* (H)	GTTTGCTGAGGAAGACAAAAAGCC	CACTTCCATAGAGTTTGCTGATATG
*CHOP* (H)	GGAAACAGAGTGGTCATTCCC	CTGCTTGAGCCGTTCATTCTC
*Bax* (H)	GAGCGAGTGTCTCCGGCGAATT	GCCACAAAGATGGTCACTGTCTGC
*Bcl-2* (H)	TGGGATGCCTTTGTGGAACTAT	GCTGATTTGACCATTTGCCTGA
*NLRP3* (H)	GATCTTCGCTGCGATCAACAG	CGTGCATTATCTGAACCCCAC
*TXNIP* (H)	GCCACACTTACCTTGCCA AT	TTGGATCCAGGAACGCTA AC
*TNF-α* (H)	ACCTCTCTCTAATCAGCCCTCT	GGGTTTGCTACAACATGGGCTA
*IL-6* (H)	ACTCACCTCTTCAGAACGAATTG	CCATCTTTGGAAGGTTCAGGTTG
*MnSOD* (H)	CGTGCTCCCACACATCAATC	TGAACGTCACCGAGGAGAAG
Catalase (H)	GCAGATACCTGTGAACTGTC	GTAGAATGTCCGCACCTGAG
*GAPDH* (H)	AACGTGTCAGTGGTGGACCTG	AGTGGGTGTCGCTGTTGAAGT
*GRP78* (R)	CTGGGTACATTTGATCTGACTGG	GCATCCTGGTGGCTTTCCAGCCATC
*CHOP* (R)	CCAGCAGAGGTCACAAGCAC	CGCACTGACCACTCTGTTTC
*TXNIP* (R)	CTGATGGAGGCACAGTGAGA	CTCGGGTGGAGTGCTTAGAG
*NLRP3* (R)	CAGACCTC CAAGACCACGACTG	CATCCGCAGCCAATGAACAGAG
*Bax* (R)	GCGAATTGGAGATGAACTGG	GTGAGCGAGGCGGTGAGGAC
*Bcl-2* (R)	CTGGTGGACAACATCGCTCTG	GGTCTGCTGACCTCACTTGTG
*GAPDH* (R)	GCACCGTCAAGGCTGAGAAC	TGGTGAAGACGCCAGTGGA

### Western blot

SV-HUC-1 cells were treated with siTXNIP, TXNIP and corresponding control plasmid, and then cultured in an incubator for 48 h. Total proteins were collected by RIPA (Cell Signaling Technology, Inc., Danvers, MA, U.S.A.). BCA Protein Assay Kit (Pierce) was used to measure the concentration of proteins, which was adjusted to 6 μg/μl using 1× loading and DEPC water. Five microliters of the samples were separated by 10% SDS/PAGE gels and then transferred to polyvinylidene fluoride membrane (PVDF, Millipore, U.S.A.). After being blocked in 5% nonfat milk in PBST (0.1% Tween 20 in PBS) for 1 h, the membrane was probed with primary antibody overnight at 4°C, washed with PBST three times and then incubated with secondary antibody (horseradish peroxidase (HRP)–conjugated goat anti-mouse/rabbit IgG, 1:2000; sc-516102/sc-2357; Santa Cruz Biotechnology, Inc. Dallas, TX, U.S.A.) at room temperature. After 2 h of incubation, the membrane was washed by PBST three times. A developer (EZ-ECL kit; Biological Industries BI) was used for development, and gray value of the strips were analyzed and counted by ImageJ (version 5.0; Bio-Rad, Hercules, CA, U.S.A.). The antibodies used were anti-GAPDH (mouse; 1:1000; ab8245; abcam), anti-GRP78 (rabbit; 1:1000; ab21685; abcam), anti-CHOP (mouse; 1:1000; ab11419; abcam), anti-NLRP3 (rabbit; 1:1000; ab214185; abcam) anti-TXNIP (rabbit; 1:1000; ab188865; abcam), anti-Bax (rabbit; 1:1000; ab32503; abcam), anti-Bcl-2 (rabbit; 1:1000; ab59348; abcam), anti-Cleaved Caspase-3 (rabbit; ab2302; abcam), anti-Catalase (rabbit; ab16731; abcam) and anti-MnSOD (rabbit; 1:1000; ab13533; abcam).

### Reagents

Ketamine and ELISA for determining IL-1β and IL-18 were purchased from Sigma, and small interfering TXNIP or NC siRNA were obtained from GenePharma (Shanghai, China).

### Statistical analysis

Statistical analysis was performed using statistical software Prism 6 (GraphPad Software, Inc., San Diego, CA, U.S.A.). Data were presented as SEM. *t* test was used to detect the difference between two groups. *P*<0.05 indicated a significant difference.

## Results

### ER stress and NLRP3/TXNIP were activated by ketamine

**Figure 1:**
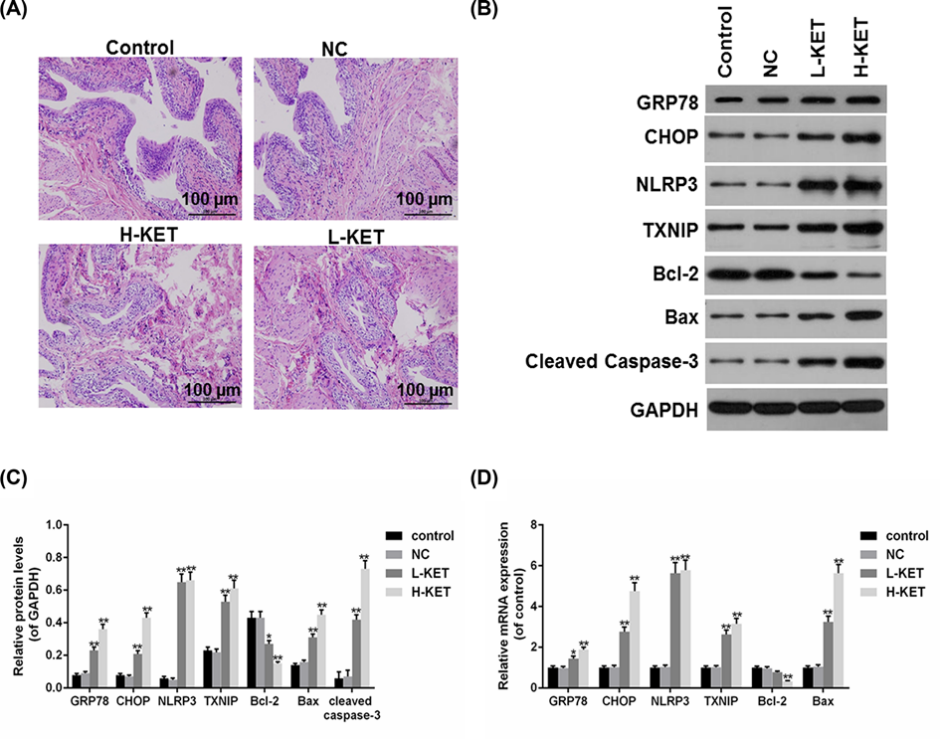
ERS-induced apoptosis and NLRP3/TXNIP were activated by ketamine (**A**) The bladder tissues of rats treated with lower and higher doses of ketamine were stained by HE staining. (**B,C**) The protein levels of GRP78, CHOP, Bax, Bcl-2, cleaved caspase-3, NLRP3 and TXNIP were determined (B) and quantified (C) by Western blot. (**D**) The mRNA levels of GRP78, CHOP, Bcl-2, Bax, NLRP3 and TXNIP were determined by RT-qPCR. *P<0.05, **P<0.01 vs. control.

A rat model of ketamine-associated cystitis was established using intraperitoneal injection of ketamine (L-KET, 5 mg/kg and H-KET, 50 mg/kg). Hematoxylin and Eosin (HE) staining results showed that rats injected with ketamine exhibited obvious edema, vascular congestion and leukocyte infiltration in a dose-dependent manner (Figure 1A). Furthermore, the levels of ER stress-induced apoptosis-associated proteins and NLRP3/TXNIP were determined by RT-qPCR and Western blot, and cleaved Caspase-3 expression was detected by Western blot. The results of Western blot demonstrated that the protein levels of GRP78, CHOP, Bax, cleaved caspase-3, NLRP3 and TXNIP were significantly increased, however, Bcl-2 protein level was decreased by ketamine (Figure 1B,C). Consistent with the protein levels results, the mRNA levels of GRP78, CHOP, Bax, NLRP3 and TXNIP were as well enhanced, while Bcl-2 was reduced in ketamine groups, compared with control and NC groups (Figure 1D).

### Cell apoptosis and NLRP3/TXNIP were promoted by ketamine in SV-HUC-1 cells

**Figure 2:**
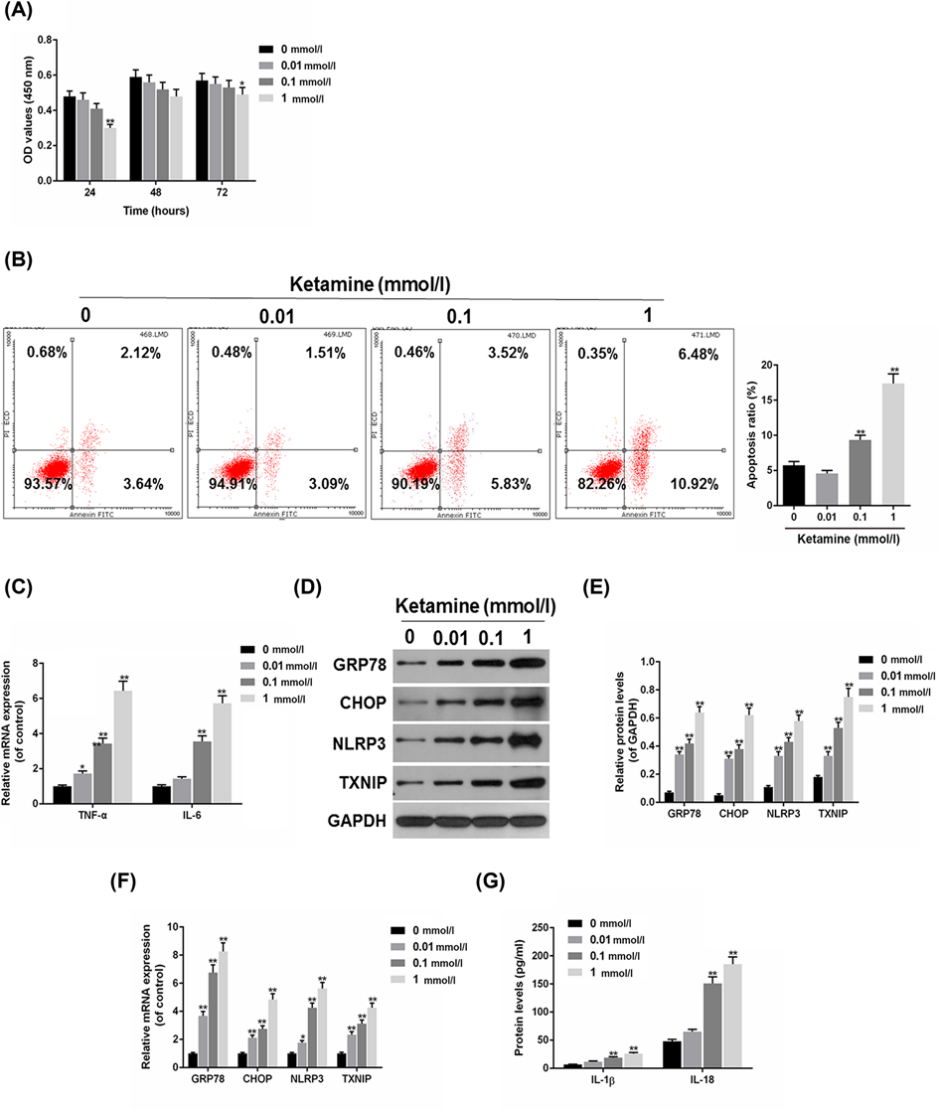
Cell apoptosis and NLRP3/TXNIP were induced by ketamine in SV-HUC-1 cells (**A**) The viability of SV-HUC-1 cells treated with 0.01, 0.1 and 1 mmol/l ketamine for 24, 48 and 72 h were determined by CCK-8. (**B**) Ketamines at 0.01, 0.1 and 1 mmol/l was used to treat SV-HUC-1 cells apoptosis, which was analyzed by flow cytometry. (**C**) TNF-α and IL-6 mRNA levels were analyzed by RT-qPCR. (**D,E**) The protein levels of GRP78, CHOP, NLRP3 and TXNIP were determined (D) and quantified (E) by Western blot. (**F**) The mRNA levels of GRP78, CHOP, NLRP3 and TXNIP were determined by RT-qPCR. (**G**) The protein levels of IL-1β and IL-18 were measured by ELISA. *P<0.05, **P<0.01 vs. control.

We found that ketamine could induce ERS-induced apoptosis and NLRP3/TXNIP *in vitro*. Furthermore, the role of ketamine in SV-HUC-1 cells was investigated. To study the effect of different concentrations of Ketamine on SV-HUC-1 cells viability, the SV-HUC-1 cells were respectively treated with 0.01, 0.1 and 1 mmol/l Ketamine for 24, 48 and 72 h. The results of CCK-8 assay indicated that cell viability was decreased by ketamine treatment in a dose-dependent manner, the viability of 1 mmol/l Ketamine treated cells decreased significantly (Figure 2A). Flow cytometry results revealed that SV-HUC-1 cells apoptosis was improved as the concentration of ketamine increased (Figure 2B). Inflammatory factors tumor necrosis factor-α (TNF-α) and IL-6 were detected using RT-qPCR, and the results showed that TNF-α and IL-6 mRNA levels were higher in ketamine groups than that in control group (Figure 2C). The expressions of GRP78, CHOP, NLRP3 and TXNIP found up-regulated in ketamine-treated SV-HUC-1 cells at protein (Figure 2D,E) and mRNA levels (Figure 2F), which were similar to the data in ketamine-treated rats (Figure 1B–D). Furthermore, ELISA found that the protein levels of IL-1β and IL-18 were improved by ketamine (Figure 2G).

### Knockdown of TXNIP inhibited SV-HUC-1 cells apoptosis and inflammation

**Figure 3:**
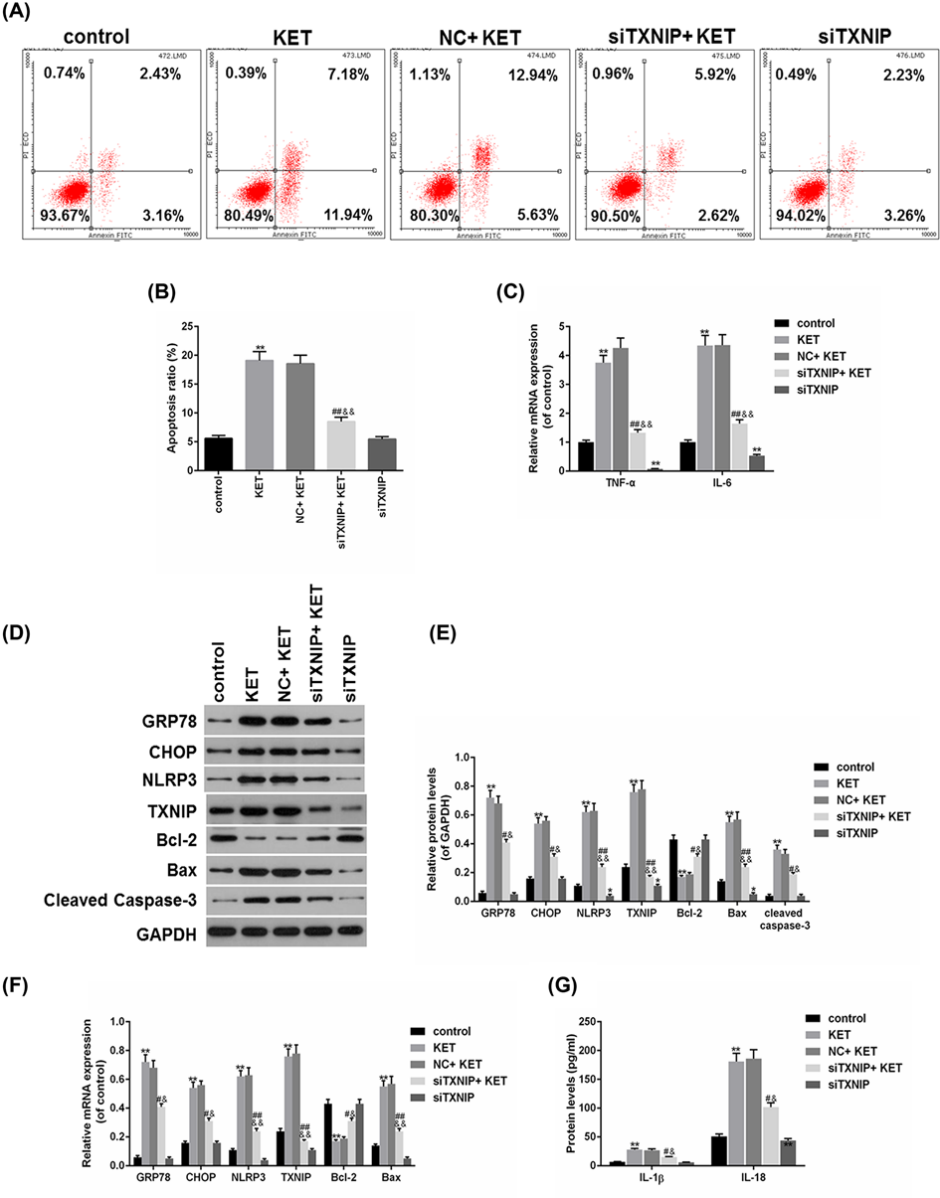
Knockdown of TXNIP inhibited SV-HUC-1 cells apoptosis and inflammation (**A,B**) SV-HUC-1 cells apoptosis was analyzed (A) and quantified (B) by flow cytometry. (**C**) TNF-α and IL-6 mRNA levels were analyzed by RT-qPCR. (**D,E**) The protein levels of GRP78, CHOP, Bax, Bcl2, cleaved caspase-3, NLRP3 and TXNIP were determined (D) and quantified (E) by Western blot. (**F**) The mRNA levels of GRP78, CHOP, Bax, Bcl2, NLRP3 and TXNIP were determined by RT-qPCR. (**G**) The protein levels of IL-1β and IL-18 were measured by ELISA. *P<0.05, **P<0.01 vs. control, ^#^*P*<0.05, ^##^*P*<0.01 vs. KET, ^&^*P*<0.05, ^&&^*P*<0.01 vs. NC+KET.

In order to investigate the role of TXNIP in ketamine-treated SV-HUC-1 cells, the expression of TXNIP was down-regulated by small interfering RNA. By performing functional experiments, we found that ketamine induced apoptosis, which could be reversed by down-regulating TXNIP (Figure 3A,B). Knockdown of SOX5 reversed the increase in TNF-α and IL-6 induced by ketamine (Figure 3C). In addition, the protein levels of GRP78, CHOP, Bax, cleaved caspase-3, NLRP3 and TXNIP were significantly lower, while Bcl-2 was higher in siTXNIP+KET group than that in NC+KET group (Figure 3D,E). Meanwhile, the data on mRNA levels of GRP78, CHOP, Bax, Bcl-2, NLRP3 and TXNIP were consistent with the Western blot results (Figure 3F). Moreover, down-regulation of TXNIP partially reversed the protein levels of IL-1β and IL-18 under the effect of ketamine (Figure 3G).

### Knockdown of TXNIP reversed ROS production caused by ketamine in SV-HUC-1 cells

**Figure 4:**
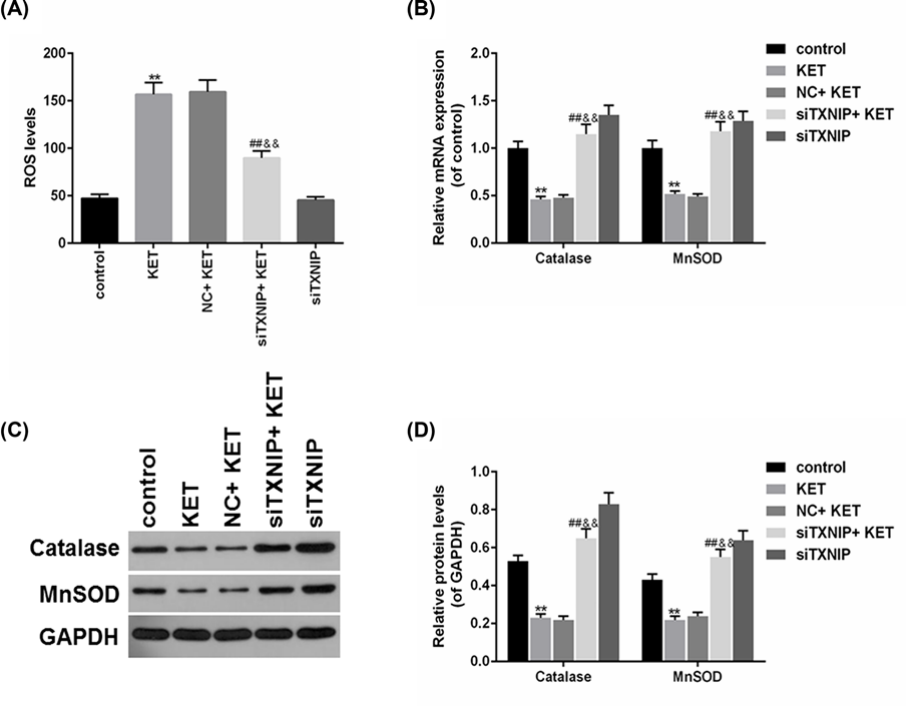
Knockdown of TXNIP reversed ROS production caused by ketamine in SV-HUC-1 cells (**A**) SV-HUC-1 cells ROS production was analyzed by flow cytometry. (**B,C**) The protein levels of Catalase and MnSOD were determined (B) and quantified (C) by Western blot. (**D**) The mRNA levels of Catalase and MnSOD were assessed by RT-qPCR. **P<0.01 vs. control, ^##^*P*<0.01 vs. KET, ^&&^*P*<0.01 vs. NC+KET.

To study the effect of TXNIP on ROS production in Ketamine-treated SV-HUC-1 cells, ROS and antioxidant gene (Catalase and MnSOD) were detected using flow cytometry, RT-qPCR and Western blot, respectively. The results of flow cytometry showed that ROS production was obviously increased by ketamine, which could be ameliorated by knocking down TXNIP (Figure 4A). Furthermore, the Catalase and MnSOD protein levels were found increased in siTXNIP + KET in comparison with NC+KET group (Figure 4B,C), and the mRNA levels of Catalase and MnSOD were decreased by Ketamine, which could be reversed by down-regulating TXNIP (Figure 4D).

## Discussion

In the present study, we investigated the underlying mechanisms of ketamine-associated cystitis *in vivo* and *in vitro* using Wistar rats and SV-HUC-1 cells. Our data suggested that ketamine damaged rat bladder tissues and induced ERS-related apoptosis and TXNIP/NLRP3 signal pathway *in vivo*. Furthermore, we also found that ketamine increased ERS-related apoptosis, ROS production and TXNIP/NLRP3 signal pathway in SV-HUC-1 cells. However, these phenomena by ketamine on SV-HUC-1 cells could be ameliorated when the SV-HUC-1 cells were transfected with small interfering RNA targeting at TXNIP.

Evidence showed that a long-term abuse of ketamine could affect urinary system and result in ketamine-associated cystitis [23–25]. For example, a study reported that severe urinary frequency was induced by a long-term abuse of ketamine in rats [26]. Kidney dysfunction including proteinuria, fibrosis of the urinary bladder and reduction in size of the urinary bladder leading to frequent urination were observed in patients who had a long history of ketamine use [23]. However, the pathogenesis of ketamine-associated cystitis is still poorly understood. In our study, we used Wistar rats and SV-HUC-1 cells to build a model of ketamine-associated cystitis by ketamine treatment. The results indicated that ketamine treatment caused significant damages to rat bladder tissues and reduced SV-HUC-1 cell viability, and these phenomena were similar to those in the patients and mice who had a long history of taking ketamine [27,28]. Those data suggested that the model of ketamine-associated cystitis *in vivo* and *in vitro* were successfully established and laid the foundation to further research on the underlying mechanisms.

It has been reported that ketamine could induce abnormal expression of ER stress-related proteins in nerve cells, liver cells and renal tubular epithelial cells [29,30]. ER stress will be activated by the increase in CHOP/GADD153 mRNA levels in adult neural stem cells in exposure to ketamine [30]. A study found that after ketamine treatment, the expressions of caspase-3, caspase-8, caspase-9, cytochrome *c* and PARP as well as TUNEL-positive cells were significantly increased in bladder tissue [31]. Consistent with above studies, we found that ER stress-related protein including GRP78 and CHOP were increased when the rats and the SV-HUC-1 cells were treated with ketamine. CHOP is a transcriptional regulator and an essential regulator of ER stress-mediated apoptosis pathway [32]. This study revealed that anti-apoptosis protein Bcl2 was down-regulated while pro-apoptosis proteins (Bax and cleaved caspase 3) were up-regulated in ketamine-treated rats. Meanwhile, SV-HUC-1 cells apoptosis was significantly enhanced after exposed SV-HUC-1 cells to ketamine. Those results indicated that ketamine-activated ER stress and thereby induced cell apoptosis, which may be one of the mechanisms of ketamine-associated cystitis.

It is recognized that overproduction of ROS and inflammation could impair cellular functions, resulting in cell death [33,34]. A study reported that the levels of NLRP3, IL-1β and IL-18 were significantly up-regulated after using multiple doses of ketamine to induce ketamine-related neurotoxicity in the hippocampus in neonatal and juvenile mouse [35]. It has been proved that TXNIP was related to ROS and NLRP3 inflammasome activation [36]. Recent studies suggested that ketamine increased the ROS production in primary hippocampal neurons [37,38]. Here, our results observed that ketamine increased TXNIP and NLRP3 levels *in vivo* and *in vitro*. Furthermore, the expressions of IL-1β, IL-18, TNF-α and IL-6 were elevated in ketamine-treated SV-HUC-1 cells, in which, ROS production was increased as well, accompanied with decreased Catalase and MnSOD levels. To investigate the important role of TXNIP in ketamine-associated cystitis, the expression of TXNIP was knocked down by small interfering RNA. By conducting functional experimentals, we discovered that the effects of ketamine on apoptosis, ER stress-correlated proteins and NLRP3 inflammasome and ROS in SV-HUC-1 cells were reversed by the down-regulation of TXNIP. These data demonstrated that TXNIP/NLRP3 aix and ROS participated in the progression of ketamine-associated cystitis at least in rats and SV-HUC-1 cells.

There are limitations in the present study, no in-depth study was conducted on the defined times of ketamine injection or treatment in rat and cells, considering that ketamine abusers would experience long duration of ketamine injection. Beyond that, there was also a lack of clinical data in our experiments, though clinical data have shown that ketamine abusers would suffer ketamine-associated cystitis. In addition, more elaborate studies will be necessary for further exploration of the role of ketamine on cystitis. Whether the activation of NLRP3 will affect the pyrogeny of SV-HUC-1 cells needs further discussion.

## Conclusion

In conclusion, we revealed that in rat and cells models, ketamine-associated cystitis was associated with ER stress, apoptosis, TXNIP/NLRP3 aix and ROS. Our study provided a deeper understanding and a treatment strategy for ketamine-associated cystitis.

## Abbreviations

Bax: Bcl-2-associated X protein
Bcl2: B-cell lymphoma/leukemia-2
CCK-8: cell counting kit-8
cDNA: complementary deoxyribonucleic acid
CHOP: CCAAT/enhancer binding protein homologous protein
DEPC: diethylpyrocarbonate
ELISA: enzyme-linked immunosorbent assay
ER: endoplasmic reticulum
ERS: endoplasmic reticulum stress
GADD153: growth arrest and DNA damage-inducible gene153
GAPDH: glyceraldehyde-3-phosphate dehydrogenase
GRP78: glucose-regulated protein 78
IL: interleukin
NC: negative control
NLRP3: NOD-like receptor 3
OD: optical density
PARP: poly ADP-ribose polymerase
PBS: phosphate-buffered saline
PBST: 0.1% Tween 20 in PBS
PI: propidium iodide
qPCR: quantitative polymerase chain reaction
ROS: reactive oxygen species
RT-qPCR: Real-time quantitative polymerase chain reaction
TNF-α: tumor necrosis factor-α
TUNEL: Terminal deoxynucleotidyl transferase-mediated dUTP nick-end labeling
TXNIP: thioredoxin-interacting protein
